# A population of naive‐like CD4^+^ T cells stably polarized to the T_H_1 lineage

**DOI:** 10.1002/eji.202149228

**Published:** 2022-02-12

**Authors:** Jonathan W. Lo, Maria Vila de Mucha, Stephen Henderson, Luke B. Roberts, Laura E. Constable, Natividad Garrido‐Mesa, Arnulf Hertweck, Emilie Stolarczyk, Emma L. Houlder, Ian Jackson, Andrew S. MacDonald, Nick Powell, Joana F. Neves, Jane K. Howard, Richard G. Jenner, Graham M. Lord

**Affiliations:** ^1^ School of Immunology and Microbial Sciences King's College London London UK; ^2^ Division of Digestive Diseases Faculty of Medicine Imperial College London London UK; ^3^ UCL Cancer Institute and CRUK UCL Centre University College London (UCL) London UK; ^4^ School of Life Sciences, Pharmacy and Chemistry Kingston University London UK; ^5^ Abcam Plc. Cambridge Biomedical Campus Cambridge UK; ^6^ School of Cardiovascular Medicine and Sciences Guy's Campus, King's College London London UK; ^7^ Lydia Becker Institute of Immunology and Inflammation, Faculty of Biology, Medicine and Health University of Manchester Manchester UK; ^8^ Centre for Host‐Microbiome Interactions King's College London London UK; ^9^ School of Biological Sciences, Faculty of Biology, Medicine and Health University of Manchester Manchester UK

**Keywords:** CD4 T cells, Gene regulation, Immune regulation, T helper cells, Transgenic models, T cells, T‐bet, naive T cells, TH1, mouse model, colitis, IFNg

## Abstract

T‐bet is the lineage‐specifying transcription factor for CD4^+^ T_H_1 cells. T‐bet has also been found in other CD4^+^ T cell subsets, including T_H_17 cells and Treg, where it modulates their functional characteristics. However, we lack information on when and where T‐bet is expressed during T cell differentiation and how this impacts T cell differentiation and function. To address this, we traced the ontogeny of T‐bet‐expressing cells using a fluorescent fate‐mapping mouse line. We demonstrate that T‐bet is expressed in a subset of CD4^+^ T cells that have naïve cell surface markers and transcriptional profile and that this novel cell population is phenotypically and functionally distinct from previously described populations of naïve and memory CD4^+^ T cells. Naïve‐like T‐bet‐experienced cells are polarized to the T_H_1 lineage, predisposed to produce IFN‐γ upon cell activation, and resist repolarization to other lineages in vitro and in vivo. These results demonstrate that lineage‐specifying factors can polarize T cells in the absence of canonical markers of T cell activation and that this has an impact on the subsequent T‐helper response.

## Introduction

Upon encounter with specific antigen, naïve CD4^+^ T cells differentiate into one of several T_H_ cell subtypes, including T_H_1, T_H_2, T_H_17, follicular T cells (T_FH_), and peripheral regulatory T cells (pTreg). CD4^+^ T cell fate depends on the cytokine environment, signaling through the TCR, and the transcription factors that these stimuli induce. Differentiation of T_H_1 cells is triggered in response to presentation of bacterial antigens by APCs [1] and requires IL‐12‐mediated activation of STAT4 [2]. T_H_1 cells are characterized by their production of IFN‐γ, which activates cell‐mediated immunity against intracellular bacteria, viruses, and tumor cells. Inappropriate or excessive T_H_1 responses also contribute to inflammatory diseases, highlighting the importance of understanding the regulation of T_H_1 cell differentiation.

Differentiation of naïve T cells into T_H_1 effectors requires the T‐box transcription factor T‐bet, encoded by *Tbx21*, which is upregulated via IL12‐dependent activation of STAT4 [2, 3]. T‐bet directly activates *Ifng*, and a number of other genes encoding cytokines and receptors including *Il12rb2*, *Cxcr3* and *Ccl4* [2, 4‐6]. T‐bet also activates its own expression, by both directly binding to its own gene and via IFN‐γ‐mediated activation of STAT1 [7, 8]. T‐bet mediated *Ifng* activation is accompanied by chromatin modifications that are maintained in memory cells [9, 10, 11]. In addition to promoting T_H_1 lineage‐specification, T‐bet also counteracts differentiation of CD4^+^ T cells into other lineages. T‐bet represses expression of the gene encoding the T_H_2 lineage‐specifying factor *Gata3* [12] and blocks GATA3 binding to its target genes [8, 13]. T‐bet also supresseses T_H_17 differentiation by blocking upregulation of *Rorc* and *Irf4* [14, 15].

Although serving as the T_H_1 lineage‐specifying transcription factor, under certain conditions, T‐bet can also be expressed in T_H_2, T_H_17, pTreg, and in a T_FH_‐T_H_1 transitional state, revealing a level of plasticity in CD4^+^ T‐cell lineage specification [16, 17, 18]. Functionally, T‐bet expression in T_H_17 cells is associated with disease in murine allergic encephalomyelitis [19, 20, 21] and T‐bet is required for T_reg_ homoeostasis and function during T_H_1‐mediated inflammation [22]. T‐bet also plays key roles outside of CD4^+^ T cells, including CD8^+^ cytotoxic T cells, γδ T cells, NK cells, NKT cells, type I and type 3 innate lymphoid cells (ILC1s and ILC3s), DCs, monocytes, and B cells [3].

Dysregulated T‐bet function is associated with inflammatory disease. T‐bet is upregulated in lamina propria CD4^+^ T cells of patients with Crohn's and celiac disease and ex vivo culture of biopsies from untreated celiac patients with gliadin peptides increases T‐bet expression through STAT1 activation [23, 24]. Genetic variants associated with ulcerative colitis and Crohn's disease are enriched at T‐bet binding sites and can alter T‐bet binding to its target genes [25]. Transfer of *Tbx21^–/–^* naïve CD4^+^ T cells into *Rag2^–/–^* mice gives rise to a higher proportion of IL‐17A‐producing CD4^+^ T cells and more severe colitis compared to mice receiving WT naïve CD4^+^ T cells [15, 26], suggesting that T‐bet restrains pathology in IL‐17‐driven colitis [26, 27, 28, 29].

Naïve CD4^+^ T cells can be identified by the surface markers CD62L^high^ CD44^low^ CCR7^high^ CD28^+^ CD27^+^. Consistent with their presumed non‐antigen experienced state, CD4^+^ T cells with these markers are not considered to express lineage‐specifying transcription factors, apart from GATA3, which is essential for CD4^+^ T cell homeostasis, in addition to its role in T_H_2 lineage‐specification. In contrast, antigen‐experienced central memory T cells (T_CM_) and effector memory T cells (T_EM_) are defined as CD62L^high^ CD44^high^ CCR7^high^ CD28^+^ CD27^int^ and CD62L^low^ CD44^high^ CCR7^low^ CD28^+^ CD27^–^, respectively [30, 31, 32, 33, 34, 35, 36]. However, the distinction between the naïve and memory cell states is not always clear cut. Small numbers of memory‐phenotype (MP) cells are found in normal, non‐immunised mice [37, 38, 39]. MP cells, which are predominantly CD8^+^, also arise in mice maintained under germ‐free and antigen‐free conditions and in humans before birth, indicating they develop in response to self‐antigens [40, 41]. Within the MP cell population, virtual memory (VM) T cells (CD8^+^ CD62L^low^ CD44^high^ CD122^high^ CXCR3^high^ Ly6C^high^ CD49d^low^) are highly proliferative and provide both antigen‐specific immunity and exhibit bystander killing activity [40, 42, 43]. Memory T cells with a naïve phenotype (T_MNP_) are a human CD8^+^ T cell population defined as CXCR3^+^ CD49d^+^ CCR7^+^ CD95^lo^ CD28^int^ and are also highly responsive to virus peptides [44]. T_H_1‐like memory phenotype (T_H_1‐like MP) T cells (CD4^+^ CD62L^–^ CD44^+^ CXCR3^+^ ICOS^+^ CD49d^high^) express high levels of T‐bet and are rapidly able to produce IFN‐γ without the need for TCR activation [45]. Like naïve T cells, CD8^+^ T‐memory stem cells (T_SCM_) are CD62L^high^ CD44^low^ and exhibit high proliferative capacity, but co‐express CD122 (IL‐2Rβ), CXCR3, Sca‐1, Bcl2, and low levels of T‐bet [46, 47, 48]. A population of MP CD4^+^ cells is present in germ‐free and antigen‐experienced mice and developed from CD5^hi^ naïve cells in the periphery in a TCR and CD28‐dependent manner [49, 50]. These MP cells contain T‐bet^lo^, T‐bet^int^, and T‐bet^hi^ subpopulations, with T‐bet^hi^ MP cells providing rapid, nonantigen specific, upregulation of IFN‐γ in response to infection [49].

Knowledge of the CD4^+^ T cell subsets that express T‐bet, the points during T cell ontogeny at which T‐bet is expressed, and the impact of T‐bet on cell function is important for understanding T cell differentiation and its dysregulation in disease. However, progress has been limited by the challenge of identifying cells that have experienced T‐bet expression. To address this, we have developed a T‐bet^cre^ × Rosa26‐YFP^fl‐STOP‐fl^ mouse line in which YFP marks cells that have expressed T‐bet during their ontogeny. Using this line, we report the discovery of a previously unidentified CD4^+^ T cell population that has a naïve cell‐surface phenotype which, nevertheless, has experienced T‐bet expression. Although displaying naïve cell‐surface markers and transcriptional profile, these T‐bet‐experienced cells were polarized toward the T_H_1 lineage, predisposed to rapidly upregulate IFN‐γ upon activation, and stably maintained their phenotype in opposing polarization conditions *in vitro* and in an *in vivo* colitis model. This work reveals polarization of CD4^+^ T cells in the absence of canonical markers of T‐cell activation and that this shapes subsequent immune responses.

## Results

### **A population of T‐bet‐experienced CD4** ^+^ **T cells with a naïve surface phenotype**

We generated a fluorescent T‐bet fate mapping (T‐bet^FM^) mouse line to identify cells that have expressed T‐bet during their ontogeny. We first inserted an IRES‐Cre cassette downstream of the stop codon in exon 6 of the endogenous *Tbx21 gene* (Supporting Information Fig. S1A). These *T‐bet^cre/+^* mice were then crossed with *Rosa26^YFPfl/+^* to generate a T‐bet^FM^ line in which induction of *Tbx21* triggers *Cre* expression, removal of the *Eyfp* stop codon and, thus, YFP production (Supporting Information Fig. S1B). Activation of naïve CD4^+^ T cells *in vitro* demonstrated YFP production under Th1‐polarizing, but not Th2‐polarizing, conditions (Supporting Information Fig. S1C and D). Thus, this T‐bet^FM^ line allows identification of cells that express, or that have previously expressed, T‐bet.

**Figure 1:**
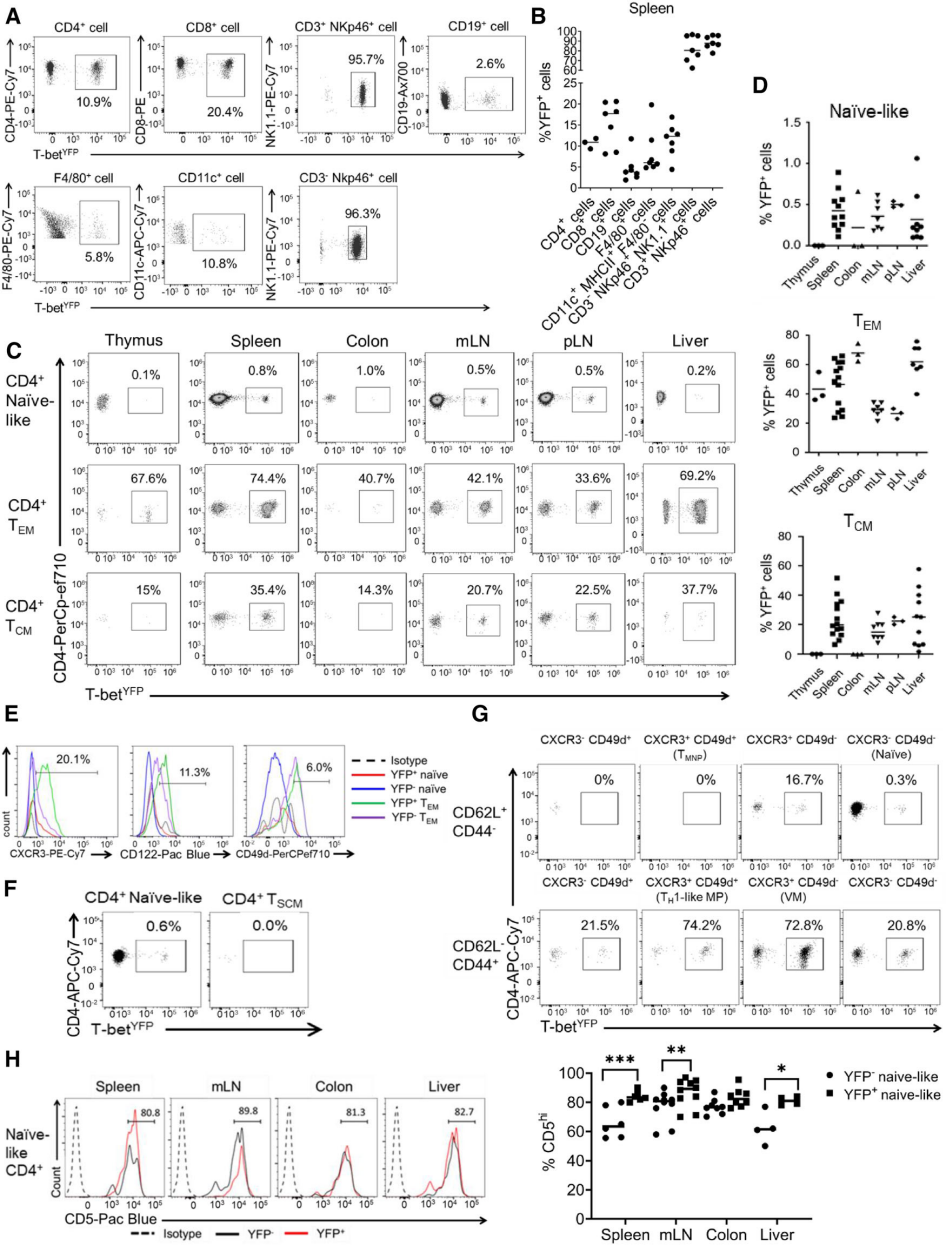
**T‐bet fate mapping identifies a population of peripheral naïve‐like CD4^+^ T cells that have experienced T‐bet expression**. (A) Representative flow plots from two independent experiments showing YFP expression in different splenic immune cells populations (n = 7 except for CD4^+^ T cells where n = 3). (B) Quantification of the mean proportions of YFP^+^ cells for each cell type shown in A (n = 7 except for CD4^+^ T cells where n = 3). (C) Representative flow plots from two independent experiments showing YFP expression in the naïve‐like (CD62L^+^ CD44^–^ CD28^+^ CD27^+^), T_CM_ (CD62L^+^ CD44^+^ CD28^+^ CD27^+^), and T_EM_ (CD62L^–^ CD44^+^ CD28^+^ CD27^–^) compartments in the thymus, spleen, colon, mesenteric lymph nodes, peripheral lymph nodes, and liver (n = 3 for thymus, colon, and pLN, n = 7 for mLN and liver, and n = 11 for spleen). (D) Mean proportions of YFP^+^ cells in the naïve, effector memory, and central memory CD4^+^ T‐cell populations shown in C (n = 3 for thymus, colon, and pLN, n = 7 for mLN and liver, and n = 11 for spleen). (E) Representative histograms from three independent experiments showing surface marker expression of CXCR3, CD122, CD49d in YFP^–^ versus YFP^+^ naïve‐like (live CD3^+^ CD4^+^ CD62L^+^ CD44^–^) and effector memory (live CD3^+^ CD4^+^ CD62L^–^ CD44^+^) CD4^+^ T cells from the spleen. Percentages of YFP^+^ naïve CD4^+^ T cells positive for each marker are shown (n = 16). (F) Representative flow plots from three independent experiments showing YFP expression by conventional naïve‐like CD4^+^ T cells (CD62L^+^ CD44^–^ CD28^+^ CD27^+^ CD127^+^ CD122^–^ CD95^–^) compared with T_SCM_ (CD62L^+^ CD44^–^ CD28^+^ CD27^+^ CD127^+^ CD122^+^ CD95^+^) from the spleen (n = 16). (G) Representative flow plots from three independent experiments showing YFP expression in splenic CD28^+^ CD27^+^ T‐cell populations divided by CD62L, CD44, CXCR3, and CD49d expression. Naïve (CD62L^+^ CD44^–^ CXCR3^–^ CD49d^–^), T_MNP_ (CD62L^+^ CD44^–^ CXCR3^+^ CD49d^+^), T_H_1‐like MP (CD62L^–^ CD44^+^ CD49d^+^ CXCR3^+^), and VM (CD62L^–^ CD44^+^ CD49d^–^ CXCR3^+^) T‐cell populations are labeled (n = 16). (H) Representative histograms and mean proportions of CD5^hi^ expressing cells from naïve‐like YFP^+^ CD4^+^ T cells (live CD3^+^ CD4^+^ CD62L^+^ CD44^–^) and naïve YFP^–^ CD4^+^ T cells in the spleen, mLN, colon, and liver from two independent experiments (n = 6 for spleen and mLN, n = 10 for colon, n = 4 for liver). Percentages shown for YFP^+^ naïve‐like CD4^+^ T cells. *p < 0.05, **p < 0.01, ***p < 0.005 (Mann–Whitney U‐test).

We first sought to validate our model by characterizing YFP expression in a basic immunophenotyping strategy for cell types that are known to express T‐bet (Supporting Information Fig. S1E). We found that 11% of CD4^+^ T cells and 18% of CD8^+^ T cells from the spleens of T‐bet^FM^ mice were YFP^+^ (Fig. 1A and B). The proportion of YFP^+^ F4/80^+^, CD11c^+^, and CD19^+^ cells in the spleen were relatively low (6, 11, and 3%, respectively), consistent with the previously characterized expression of T‐bet in subsets of these cell types [3]. In contrast, 80% of splenic CD45^+^ NKp46^+^ lineage‐negative and 90% CD45^+^ NKp46^+^ lineage‐positive cells were YFP^+^ (Fig. 1A and B), consistent with the high levels of T‐bet expression known to be exhibited by these cell types [3].

We next examined YFP expression in CD4^+^ T cells with naïve markers (CD62L^+^ CD44^–^ CD27^+^ CD28^+^), memory T_CM_ markers (CD62L^+^ CD44^+^ CD27^+^ CD28^+^), and T_EM_ markers (CD62L^–^ CD44^+^ CD27^–^ CD28^+^) [34, 35, 36] (Supporting Information Fig. S1F). This revealed that 0.5–1% of CD4^+^ T cells with a naïve surface phenotype were YFP^+^, depending on the source of the cells (Fig. 1C and D). In comparison, 15–38% of T_CM_ and 34–74% of T_EM_ cells expressed YFP (Fig. 1C and D). We conclude that a population of CD4^+^ T cells with naïve surface markers has experienced T‐bet expression.

We sought to investigate potential differences in the expression of the typical activated memory cell markers CD122, CD49d, and CXCR3 between YFP^–^ and YFP^+^ CD4^+^ naïve‐like and T_EM_ cells. We found that both YFP^–^ and YFP^+^ naïve‐like CD4^+^ T cells exhibited lower levels of CD49d and CD122 in comparison to both YFP^+^ and YFP^–^ T_EM_ populations (Fig. 1E). This further suggests that YFP^+^ naïve‐like cells are not a memory population. Interestingly, when comparing YFP^+^ versus YFP^–^ naïve‐like T cells, the only major difference in surface marker expression was CXCR3 (a T‐bet target gene), which was present on 20% of splenic YFP^+^ naïve‐like cells and absent on YFP^–^ naïve‐like CD4^+^ T cells (Fig. 1E and Supporting Information Fig. S2A). We conclude that YFP^+^ naïve‐like CD4^+^ T cells share naïve surface markers with their YFP^–^ counterparts, except for increased levels of CXCR3 and do not exhibit memory T cell markers.

### **Naïve‐like YFP‐positive cells do not correspond to previously defined CD4** ^+^ **T cell populations**

Having established that there exists a population of T‐bet experienced CD4^+^ T cells with a naïve surface phenotype, we next sought to determine the potential relationship between these cells and previously identified naïve‐like memory cell populations, in particular, memory T cell with naïve‐like phenotype (T_MNP_ cells) [44], VM T cells [40], stem cell‐like memory T cells (T_SCM_) [46, 47], T_H_1‐like memory phenotype (T_H_1‐like MP) cells [45], and memory‐phenotype (MP) cells [49].

First, we measured YFP expression in T_SCM_, which, like naïve cells, are CD62L^+^ CD44^–^ CD28^+^ CD27^+^ IL‐7R^+^, but also express the memory cell markers CD122^+^ and CD95^+^ [46, 47]. We identified very few of these cells but found that they were uniformly YFP‐negative (Fig. 1F and Supporting Information Fig. S2B). In contrast, 0.6% of the canonical naïve CD4^+^ population (CD62L^+^ CD44^–^ CD28^+^ CD27^+^ IL‐7R^+^ CD122^–^ CD95^–^) were YFP^+^ (Fig. 1F). Thus, naïve YFP^+^ CD4^+^ T cells are distinct from T_SCM_.

We next measured YFP expression in T_MNP_ cells, which, like naïve cells, are CD62L^+^ CD44^–^ CD28^+^ CD27^+^, but, unlike naïve cells are also CXCR3^+^ and CD49d^+^ [44]. However, we identified very few T_MNP_ cells within the peripheral organs of T‐bet^FM^ mice (Supporting Information Fig. S2C) and none of these cells were YFP^+^ (Fig. 1G). In contrast, 0.3% of CXCR3^–^ CD49d^–^ naïve CD4 T cells were YFP^+^ and 16.7% of CXCR3^+^ CD49d^–^ cells were also YFP^+^ (Fig. 1G). This demonstrated that YFP^+^ naïve‐like CD4^+^ T cells do not correspond to T_MNP_ cells. Using the same gating strategy, it was also possible to identify VM cells (CD62L^–^ CD44^+^ CD49^–^ CXCR3^+^) [40]. A total of 73% of these cells were YFP^+^, but since they were not CD62L^+^ and CD44^–^, these are distinct from the naïve YFP^+^ cell population (Fig. 1G).

We then examined T_H_1‐like MP cells [45], using the same gating strategy as before (Supporting Information Fig. S2C), and found that 74.2% of these cells were YFP^+^ (Fig. 1G), consistent with the prior demonstration that these cells express T‐bet and produce IFN‐γ. However, unlike naïve cells, T_H_1‐like MP cells are CD62L^–^ CD44^+^ CXCR3^+^ CD49d^+^ [45] and, thus, are distinct from the naïve‐like YFP^+^ cell population.

Naïve YFP^+^ cells were also distinct from another described population of MP cells, which are CD62L^–^ CD44^+^ CD28^+^ CD5^hi^ [49]. CD5^hi^ naive cells have been found to generate more MP cells than their CD5^lo^ counterparts in the absence of foreign antigen, suggesting an involvement of reactivity to self‐antigen in MP cell generation. Thus, we considered that the naïve‐like YFP^+^ cell population that we observed may also be CD5^hi^. We found that only a small minority of naïve CD5^hi^ cells were YFP^+^ (Supporting Information Fig. S2D) but that a higher proportion of YFP^+^ cells were CD5^hi^ compared to YFP^–^ cells (Fig. 1H), consistent with exposure to self‐antigen. We conclude that there is a population of T‐bet experienced CD4^+^ T cells with naïve surface markers that is distinct from previously described CD4^+^ cell populations.

### T‐bet‐experienced CD62L^+^ CD44^–^ CD4^+^ T cells have a naïve gene expression profile

We sought to further characterize the novel population of T‐bet experienced naïve‐like CD4^+^ T cells that we had identified in comparison to YFP^–^ naïve cells and to YFP^+^ and YFP^–^ T_EM_ compartments. We sorted YFP^+^ and YFP^–^ CD62L^+^ CD44^–^ naïve‐like and CD62L^–^ CD44^+^ T_EM_ CD4^+^ T cells and profiled their transcriptomes using RNA‐seq.

**Figure 2:**
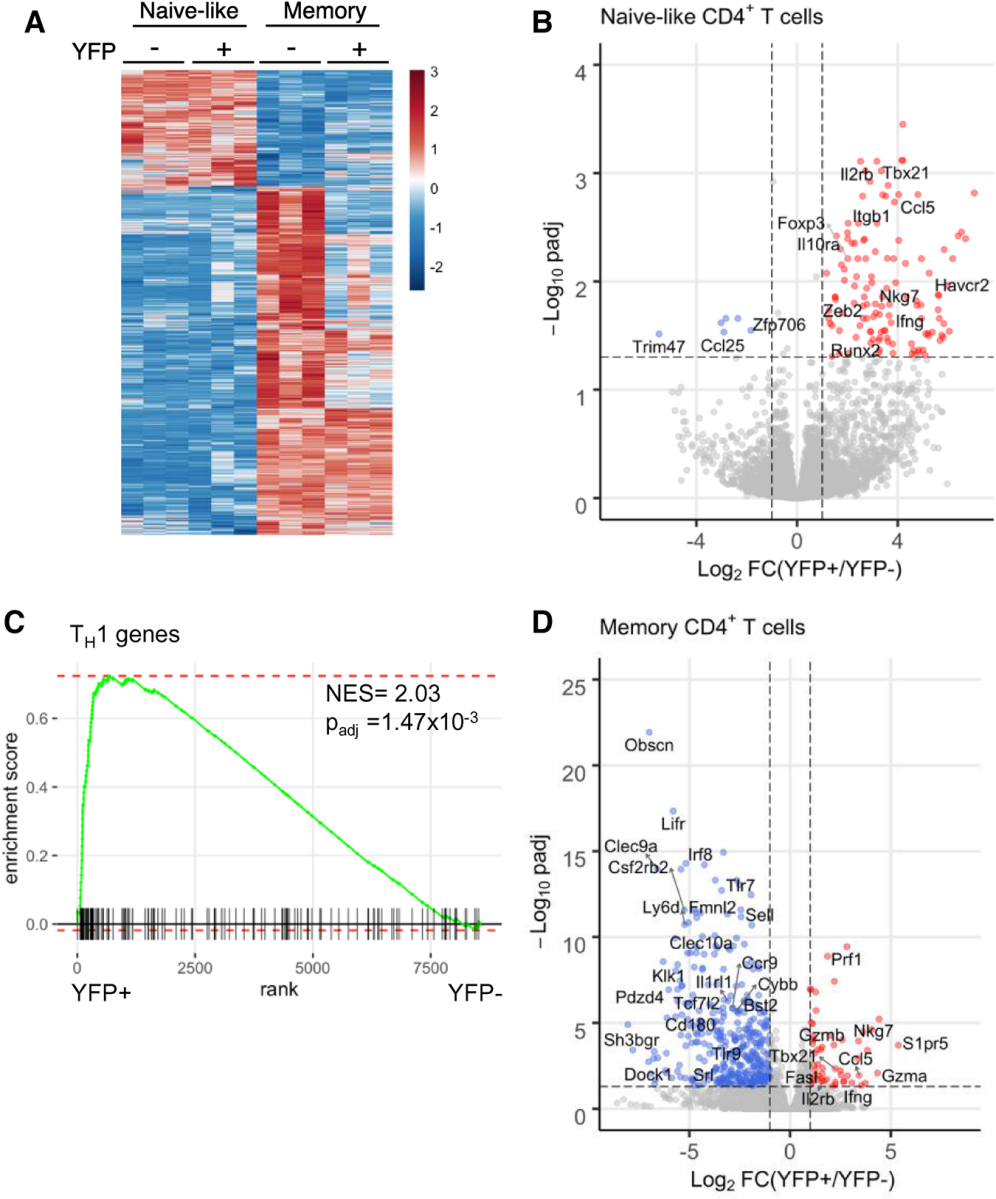
**YFP^+^ naïve‐like CD4^+^ T cells have a naïve‐like gene expression profile but exhibit Th1 polarization**. (A) Heatmap of relative gene expression between YFP^–^ naïve (live CD3^+^ CD4^+^ CD62L^+^ CD44^–^), YFP^+^ naïve‐like, YFP^–^ T_EM_ (live CD3^+^ CD4^+^ CD62L^–^ CD44^+^), and YFP^+^ T_EM_ cells (n = 3). Each row represents a gene and each column a cell sample. Gene expression is shown as *z*‐score and coloured according to the scale on the right. (B) Volcano plot showing differential gene expression between YFP^+^ and YFP^–^ naïve‐like CD4^+^ T cells (log2‐fold change vs. adjusted *p*‐value). Genes more highly expressed in YFP^+^ cells are in red and those more highly expressed in YFP^–^ cells are in blue (*p*_adj_ < 0.05, |log2FC|>1, n = 3). Selected differentially expressed genes are labeled. (C) Gene set enrichment analysis (GSEA) demonstrating enrichment of T_H_1 genes within the genes more highly expressed in naïve‐like YFP^+^ CD4^+^ cells versus naïve‐like YFP^–^ CD4^+^ cells. (D) As B, except showing differential gene expression between YFP^+^ and YFP^–^ T_EM_ CD4^+^ T cells (n = 3).

Visualizing the gene expression profiles of these cell populations revealed that YFP^+^ naïve‐like cells were more similar to their YFP^–^ naïve counterparts than to either the YFP^+^ or YFP^–^ T_EM_ cell populations (Fig. 2A). This was also apparent when visualizing gene expression between the cell populations, with YFP^+^ and YFP^–^ naïve‐like cells exhibiting differential expression of genes in comparison to both T_EM_ populations, including increased expression of *Tcf7, Lef1*, and *Sell* and decreased expression of *Cd44, Klrg1*, and *Prdm1* (Fig. 2B and Supporting Information Table S1). These data are consistent with our flow cytometric analyses showing that YFP^+^ naïve cells display naïve cell‐surface markers.

We next sought to identify the genes that distinguished YFP^+^ naïve‐like cells from YFP^–^ naïve‐like cells. We found that YFP^+^ cells exhibited higher expression of *Tbx21* and several known T‐bet target genes including *Ifng, Ccr5, Ccl5, Itgb1, Havcr2*, and *Nkg7* (Fig. 2B and Supporting Information Table S1). Furthermore, gene set enrichment analysis (GSEA) demonstrated significant enrichment of T_H_1 genes (NES = 2.03, *p*_adj_ = 1.47 × 10^–3^) within the set of genes more highly expressed in YFP^+^ versus YFP^–^ naïve cells (Fig. 2C). Thus, although the overall expression program of YFP^+^ naïve‐like cells resembles YFP^–^ naïve cells, they also display hallmarks of polarization toward a T_H_1 lineage phenotype.

We next turned our attention to the expression profile of YFP^+^ T_EM_ cells compared to their YFP^–^ counterparts. As for the naïve cells, we identified higher expression of several T‐bet target genes including *Ifng, Tbx21, Ccl5, Itga1, Fasl, Prf1, Gzmb*, and *Nkg7* (Fig. 2D and Supporting Information Table S1). We conclude that YFP^+^ naïve and memory subsets have distinct gene expression profiles from their YFP^–^ counterparts, which are consistent with T‐bet function in these cells.

### Naïve CD4^+^ T cells develop and exit the thymus without expressing T‐bet

**Figure 3:**
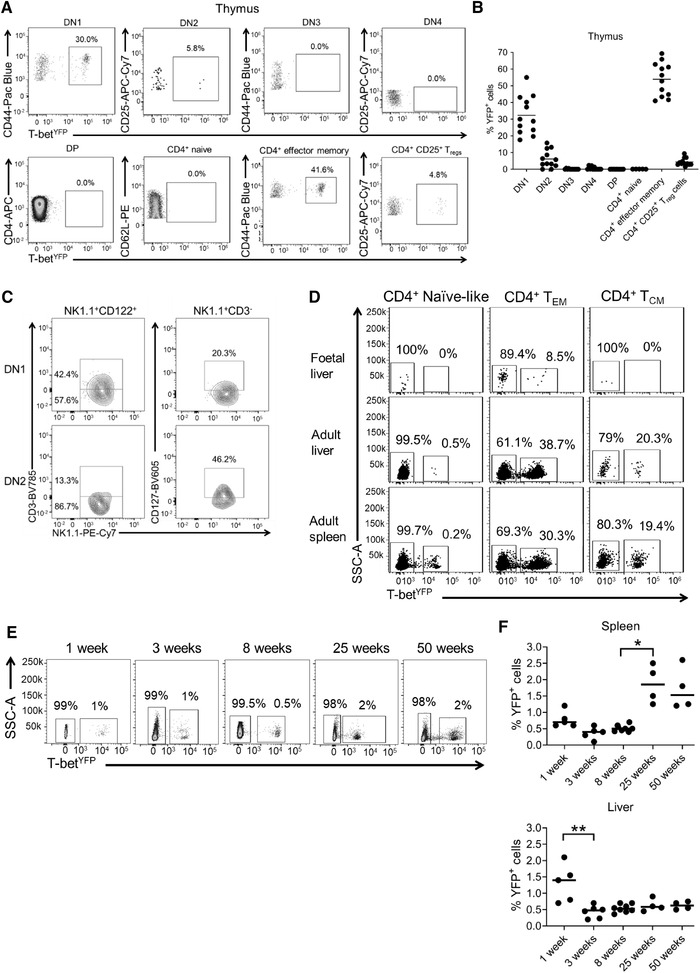
**Naïve CD4^+^ T cells develop and exit the thymus without expressing T‐bet and are not found in utero**. (A) Representative flow plots from three independent experiments with four mice in each experiment showing YFP expression in thymic DN1 (CD3^–^ CD44^+^ CD25^–^), DN2 (CD3^–^ CD44^+^ CD25^+^), DN3 (CD3^+^ CD44^–^ CD25^+^), DN4 (CD3^+^ CD44^–^ CD25^–^), DP (CD3^+^ CD4^+^ CD8^+^), naïve CD4^+^ T cells (CD3^+^ CD4^+^ CD8^–^ CD62L^+^ CD44^–^), effector memory CD4^+^ T cells (CD3^+^ CD4^+^ CD8^–^ CD62L^–^ CD44^+^) and Treg (CD3^+^ CD4^+^ CD8^–^ CD25^+^). (B) Mean proportions of YFP^+^ cells in the different thymic cell subsets shown in A (n = 12). (C) Representative flow plots from three independent experiments with four mice in each experiment showing NK and NKT markers on YFP^+^ cells from DN1 (live CD4^–^ CD8^–^ CD44^+^ CD25^–^ CD122^+^ NK1.1^+^) and DN2 (live CD4^–^ CD8^–^ CD44^+^ CD25^+^ CD122^+^ NK1.1^+^) cells from the thymus. (D) Representative flow plots from one experiment showing YFP expression in naïve‐like (CD62L^+^ CD44^–^), effector (CD62L^–^ CD44^+^) and central memory (CD62L^+^ CD44^+^) CD4^+^ T cells in the fetal liver (n = 12 fetuses pooled into one sample), adult liver (n = 6), and adult spleen (n = 6). (E) Representative flow plots showing YFP expression in splenic naïve (CD62L^+^ CD44^–^) CD4^+^ T cells from 1‐week (n = 5), 3‐week (n = 6), 8‐week (n = 8), 25‐week (n = 4), and 50‐week (n = 4) old mice. (F) Mean proportions of YFP^+^ cells in the naïve (CD62L^+^ CD44^–^) CD4^+^ T‐cell population from 1‐week (n = 5), 3‐week (n = 6), 8‐week (n = 8), 25‐week (n = 4), and 50‐week (n = 4) old mice. *p < 0.05, **p < 0.01 (Kruskal–Wallis test with Dunn's corrections).

We next sought to determine when naïve‐like CD4^+^ T cells experienced T‐bet expression during their ontogeny, beginning in the thymus. We found that thymic double‐negative (DN)3, DN4, double‐positive (DP), and CD4 single‐positive (SP) cells completely lacked YFP expression (Fig. 3A and B and Supporting Information Fig. S3A). Unexpectedly, the CD3^–^ CD44^+^ CD25^–^ and CD3^–^ CD44^+^ CD25^+^ populations exhibited a high proportion of YFP^+^ cells (32 and 5%, respectively; Fig. 3A and B). However, further immunophenotyping demonstrated that the majority (77%) of these thymic YFP^+^ cells were in fact ILC1 (CD127^+^ NK1.1^+^ CD122^+^; 35% of cells; Fig. 3C and Supporting Information Fig. S3B) or CD3^+^ NK cells (CD122^+^ NK1.1^+^ CD3e^+^; 42% of cells; Fig. 3C and Supporting Information Fig. S3B). Other YFP‐positive cells in the thymus included a small population of effector (CD62L^–^ CD44^+^) CD4^+^ T cells, of which ∼50% expressed YFP, and 5% of thymic Treg (Fig. 3A and B). However, the lack of YFP expression in DN3, DN4, DP, and CD4 SP cells shows that all mature naïve SP CD4^+^ cells leave the thymus as YFP‐negative.

We also examined YFP expression in other thymic cell populations: γδ T cells (Supporting Information Fig. 4A‐C), which diverge from αβ T cell development at the DN2‐DN3 stages, and NKT cells (Supporting Information Fig. 4D‐F), which arise from the DP stage. As expected, no progenitor γδ T cells expressed YFP (Supporting Information Fig. 4B and C). Surprisingly, however, 20% of CD24^+^ immature γδ T cells were YFP^+^, meaning that some of the cells have expressed T‐bet despite not being fully mature (Supporting Information Fig. 4B and C). As expected, a high proportion (50%) of CD25^–^CD27^+^CD24^–^ γδ T cells expressed YFP (Supporting Information Fig. 4B and C), consistent with the previous description of these cells as IFN‐γ‐producers. Interestingly, a small population (8%) of CD25^–^CD27^–^ γδ T cells, previously reported as IL‐17A‐producers, were also YFP^+^ (Supporting Information Fig. S4B and C). We conclude that as the majority of γδ thymocytes mature, there is an increment in the proportion of cells that express YFP, predisposing these cells to become T‐bet^+^ IFN‐γ^+^. Furthermore, there appears to be some maturing IL‐17A‐producer CD25^–^CD27^–^ γδ T cells that have also expressed T‐bet during development.

**Figure 4:**
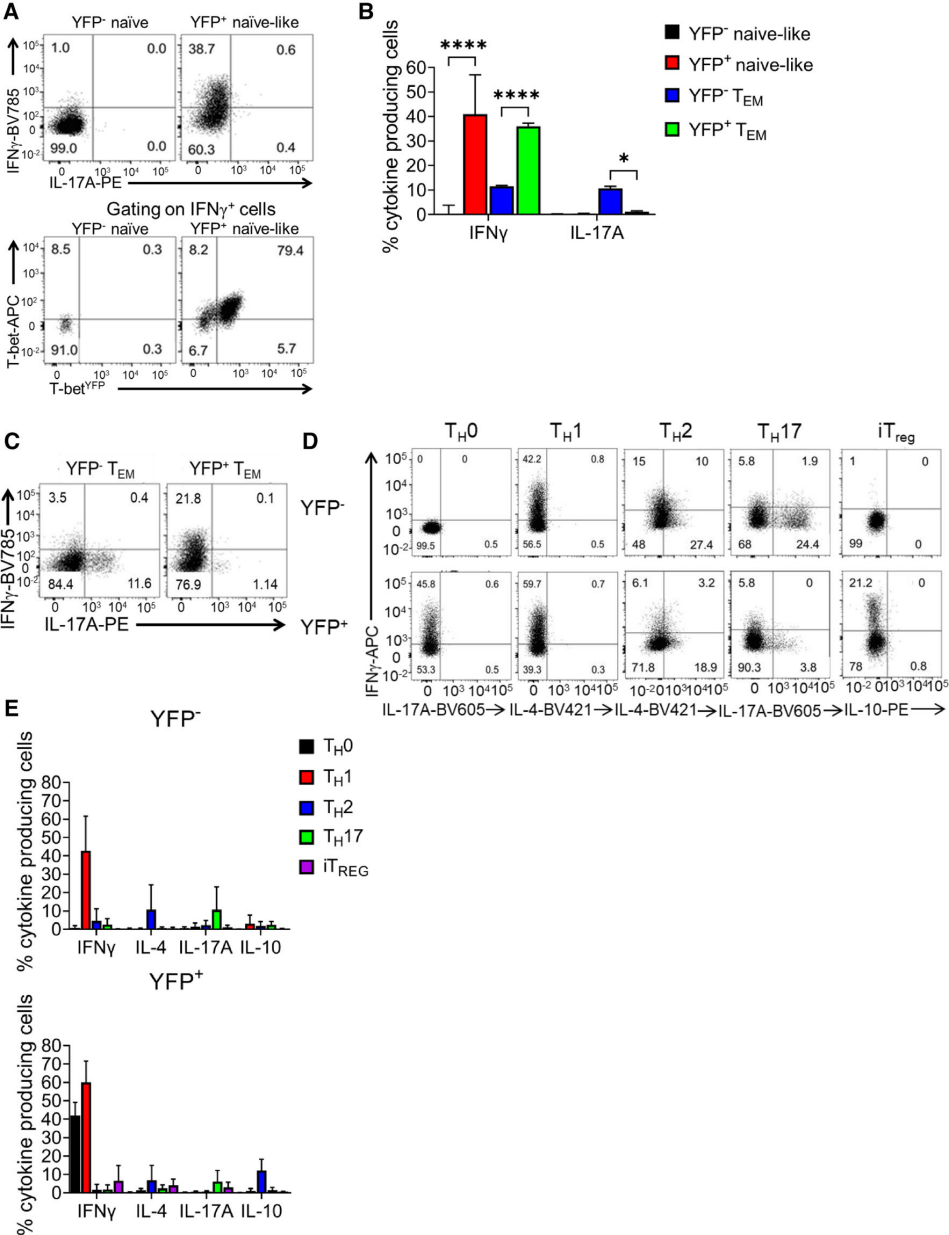
**YFP^+^ naïve‐like CD4^+^ T cells resist polarization toward other CD4^+^ T‐cell lineages**. (A) Representative flow plots from three independent experiments (each comprising three replicates) showing cytokine expression (top) or T‐bet and YFP expression (bottom) in YFP^–^ and YFP^+^ naïve‐like (CD62L^+^ CD44^–^) CD4^+^ T cells after in vitro activation with anti‐CD3/CD28 and culture with IL‐2. (B) Percentage of cultured YFP^–^ and YFP^+^ naïve‐like (CD62L^+^ CD44^–^) CD4^+^ T cells and T_EM_ (CD62L^–^CD44^+^) producing IFN‐γ or IL‐17A after activation with anti‐CD3/CD28 and culture with IL‐2 (median with range shown, n = 9), *p < 0.05, ****p < 0.0001 (Two‐way ANOVA). (C) Representative flow plots from three independent experiments (each comprising three replicates) showing cytokine expression from in vitro cultured YFP^–^ and YFP^+^ T_EM_ after activation with anti‐CD3/CD28 and culture with IL‐2. (D) Representative flow plots from three independent experiments (each comprising two replicates) showing cytokine expression in naïve‐like (CD62L^+^ CD44^–^) YFP^–^ and YFP^+^ CD4^+^ T cells after activation with anti‐CD3/CD28 and polarization in T_H_0, T_H_1, T_H_2 T_H_17 and iTreg conditions in vitro. (E) Proportion of naïve‐like (CD62L^+^ CD44^–^) YFP^–^ and YFP^+^ CD4^+^ T cells producing cytokines after activation with anti‐CD3/CD28 and polarization in T_H_1, T_H_2 T_H_17 and iTreg conditions in vitro (median with range shown, n = 6).

Next, looking at NKT cell development, we found that T‐bet expression increased with cell maturation, with immature stage 1 cells (CD44^–^ CD24^+^) completely lacking YFP expression and YFP positivity increasing to 5–10% at immature stage 2, 15–40% at immature stage 3, to finally 60–90% YFP positivity for mature NKT cells (Supporting Information Fig. S4D‐F). We conclude from this analysis that conventional CD4^+^ and CD8^+^ T cells develop in the thymus without expressing YFP and, therefore, only express T‐bet once they have left the thymus. However, NKT cells and a subpopulation of γδ T cells, which both require T‐bet to develop to maturity, show an increasing proportion of YFP^+^ cells in the thymus before leaving the thymus as mature cells.

### Naïve‐like CD4^+^ T cells become YFP^+^ shortly after birth

As we were unable to identify YFP^+^ naïve CD4^+^ T cells in the thymus, we next sought to determine whether YFP^+^ naïve CD4^+^ T cells were present before birth or only arise after birth, potentially due to exposure to environmental antigens. Examination of the fetal liver at E15.5 revealed a lack of YFP^+^ naïve or T_CM_ CD4^+^ cells, but the presence of YFP^+^ T_EM_ CD4^+^ T cells, comprising around 9% of the population (Fig. 3D). We found that by 1‐week of age, 1% of naïve‐like (CD62L^+^ CD44^–^) CD4^+^ T cells expressed YFP in the liver, and after a significant decrease at 3 weeks of age, the proportion of YFP^+^ cells in the liver remained consistent (Fig. 3E and F). A similar pattern was observed in the spleen, with around 0.6% of cells expressing YFP in 1‐week old mice, rising significantly to 1.5–2% in older animals (Fig. 3E and F). We conclude that T‐bet induction in naïve‐like T cells occurs shortly after birth. This is consistent with the gain in T‐bet expression being driven by the microbiota or other environmental factors.

### YFP^+^ naïve‐like CD4^+^ T cells are predisposed to produce IFN‐γ upon activation

We hypothesized that YFP^+^ naïve‐like cells may possess distinct properties to YFP^–^ naïve cells and that their experience of T‐bet expression may allow rapid upregulation of IFN‐γ. To test this, we purified YFP^–^ and YFP^+^ naïve‐like (CD62L^+^ CD44^–^) and T_EM_ (CD62L^–^ CD44^+^) CD4^+^ T cells, activated these cells *in vitro* in non‐polarizing conditions, and measured cytokine production and T‐bet expression by ELISA and flow cytometry. We found that activation of naïve‐like YFP^+^ cells caused robust induction of IFN‐γ that was not observed in the YFP^–^ population (Fig. 4A and B, Supporting Information Fig. S5A). In contrast, there was no production of IL‐17A, suggesting that naïve‐like YFP^+^ cells are specifically polarized toward the T_H_1 lineage. Further examination found that naïve‐like YFP^+^ cells were able to rapidly upregulate IFN‐γ, even after 3 days of culture *in vitro* in non‐polarizing conditions, when compared to naïve YFP^–^ cells (Supporting Information Fig. S5B and C). Co‐culture of CD45.2 YFP^+^ naïve‐like cells with CD45.1 naïve cells in different proportions resulted in a higher proportion of IFN‐γ‐positive CD45.2 YFP^+^ naïve‐like than IFN‐γ‐positive CD45.1 cells, demonstrating that the predisposition to produce IFN‐γ is cell autonomous (Supporting Information Fig. S5D and E).

**Figure 5:**
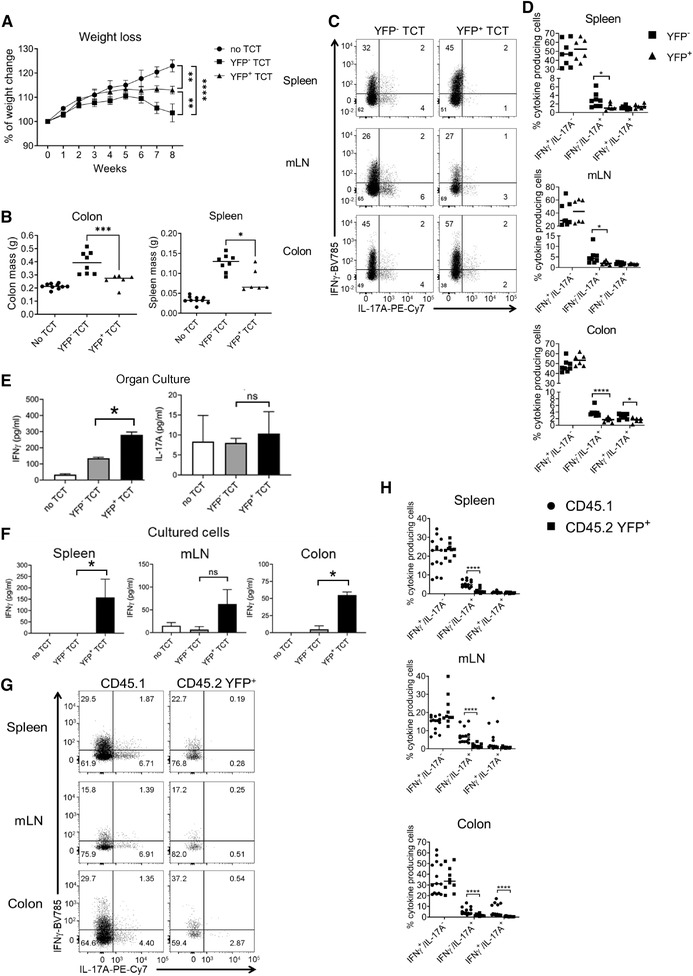
**YFP^+^ cells induce less inflammation and more IFN‐γ compared to conventional naïve CD4^+^ T cells after transfer to *Rag2^–^^/^^–^* mice**. (A) Mean weight change from two independent experiments in *Rag2^–/–^* mice after receipt of 25,000 purified naïve‐like YFP^+^ or naïve YFP^–^ CD4^+^ T cells, or no cell transfer control (n = 8 for YFP^–^ transfer, n = 6 for YFP^+^ transfer, and n = 10 for control). ** *p* < 0.01, ****p < 0.0001 (Mann‐Whitney U test performed on week 8). (B) Mean spleen and colon mass in *Rag2^–/–^* mice after receipt of 25,000 purified naïve‐like YFP^+^ or naïve YFP^–^ CD4^+^ T cells or no cell transfer control (n = 8 for YFP^–^ transfer, n = 6 for YFP^+^ transfer, and n = 10 for control.) *p < 0.05, ***p < 0.005. (Kruskal–Wallis test with Dunn's corrections). (C) Representative flow plots from two independent experiments showing the proportion of YFP^–^ and YFP^+^ naïve‐like CD4^+^ T cells that produce IFN‐γ and IL‐17A after transfer into *Rag2^–/–^* mice, separated by organ (n = 8 for YFP^–^ transfer, n = 6 for YFP^+^ transfer). (D) Mean proportion of YFP^–^ and YFP^+^ naïve‐like CD4^+^ T cells, shown in C, that produce IFN‐γ alone, IL‐17A alone, or both IFN‐γ and IL‐17A after transfer into *Rag2^–/–^* mice, separated by organ (n = 8 for YFP^–^ transfer, n = 6 for YFP^+^ transfer, from two independent experiments). *p < 0.05 ****p < 0.001 (Mann‐Whitney U test). (E) Quantification of IFN‐γ and IL‐17A in the supernatant by ELISA after 48 h of colon organ culture (n = 3 experiments, each with three technical replicates, except n = 6 experiments for the no transfer control, also with three technical replicates). *p < 0.05 (Kruskal–Wallis test with Dunn's corrections). (F) Quantification of IFN‐γ and IL‐17A in the supernatant of unfractionated cell cultures from colon, spleen, and mLN (n = 3, each with three technical replicates, except n = 6 for the no transfer control, also with three technical replicates). *p < 0.05 (Kruskal–Wallis test with Dunn's corrections). (G) Representative flow plots from two independent experiments showing IFN‐γ and IL‐17A production by naïve‐like CD45.1^+^ and CD45.2 YFP^+^ CD4^+^ T cells after cotransfer at a ratio of 9:1 (n = 8). (H) Proportions of naïve‐like CD45.1^+^ and CD45.2 YFP^+^ CD4^+^ T cells producing IFN‐γ alone, IL‐17A alone, or both IFN‐γ and IL‐17A after cotransfer at a ratio of 9:1 (n = 9). ****p < 0.001 (Mann–Whitney U test).

Consistent with T‐bet driving expression of IFN‐γ in YFP^+^ naïve‐like cells, the transcription factor was present in over 90% of IFN‐γ‐producing YFP^+^ naïve‐like cells, whereas YFP^–^ naïve cells lacked T‐bet expression (Fig. 4A). We also examined the effect of stimulation on cytokine production by YFP^+^ T_EM_ cells. As we found for their naïve‐like counterparts, YFP^+^ T_EM_ expressed IFN‐γ, but not IL‐17A, whereas YFP^–^ T_EM_ expressed either IFN‐γ or IL‐17A (Fig. 4B and C, Supporting Information Fig. S5A).

We next sought to determine whether the predisposition of YFP^+^ naïve‐like cells to produce IFN‐γ was stable under T_H_2, T_H_17, and iT_reg_ polarizing conditions. The naïve‐like YFP^+^ population resisted induction of IL‐4 or IL‐17A after culture in T_H_2‐ or T_H_17‐polarizing conditions, respectively (Fig. 4D and E). Similarly, compared to YFP^–^ naïve cells, a greater proportion of YFP^+^ naïve‐like cells maintained IFN‐γ production under T_H_2‐ and iTreg‐polarizing conditions, although induction was weaker compare to T_H_1 conditions (Fig. 4D and E). Staining for lineage‐specific transcription factors confirmed that YFP^+^ cells resisted repolarization to other lineages (Supporting Information Fig. S5F). We conclude that naïve‐like YFP^+^ cells are predisposed to express IFN‐γ and T‐bet over cytokines and transcription factors of other T‐cell lineages when polarized *in vitro*.

### **Naïve‐like YFP^+^ CD4^+^ T cells remain T_H_1 polarized in a murine T**_H_ **1/T**_H_ **17 model of colitis**

The role of T_H_1 and T_H_17 cells in inflammatory disease is reflected by the induction of colitis upon adoptive transfer of naïve CD4^+^ T cells into *Rag2^–/–^* mice, which is marked by a wasting phenotype, inflamed colon, splenomegaly, and a T_H_1/T_H_17 hybrid‐driven inflammatory response [27]. To test whether the T_H_1 polarized phenotype of YFP^+^ naïve‐like cells impacted their ability to induce colitis in this model, we transferred either 25,000 YFP^–^ or YFP^+^ CD62L^+^ CD44^–^ CD4^+^ CD25^–^ T cells into separate *Rag2^–/–^* mice and measured disease symptoms (animal, colon, and spleen weights) and cytokine production in colon organ cultures and in unfractionated cells from spleen, mesenteric lymph nodes (mLN), and colon by ELISA. We found that mice receiving YFP^–^ naïve‐like CD4^+^ T cells lost significantly more weight than mice that received YFP^+^ naïve‐like CD4^+^ T cells or mice that received no CD4^+^ T cells at all (Fig. 5A). Consistent with this, mice receiving YFP^–^ naïve‐like T cells had significantly larger colons (*p* < 0.001) and spleens (*p* < 0.05) in comparison with mice receiving YFP^+^ naïve T cells (Fig. 5B), suggesting that YFP^+^ CD4^+^ T cells induced less inflammation than their YFP^–^ counterparts.

Donor CD4^+^ T cells could be detected in the colon, mLN, and spleen after 7 weeks of transfer. Measuring cytokine production by donor cells in these organs, we found no difference in the proportion of IFN‐γ‐positive IL‐17A‐negative CD4^+^ T cells in mice receiving YFP^+^ cells versus YFP^–^ cells (Fig. 5C and D). However, the level of IFN‐γ production measured by ELISA in the supernatant from colonic biopsy organ culture (Fig. 5E) and from bulk cultures of cells from the colon (Fig. 5F) was significantly greater for mice that had received YFP^+^ naïve‐like cells. Compared to mice that received YFP^–^ cells, mice that received YFP^+^ cells displayed a significantly smaller proportion of IFN‐γ‐negative IL‐17A‐positive cells in colon, mLN, and spleen (Fig. 5C and D). Furthermore, mice that received YFP^+^ cells exhibited a significantly lower proportion of cells producing both IFN‐γ and IL‐17A in the colon (Fig. 5D). This demonstrates that the predisposition of YFP^+^ CD4^+^ T cells to produce IFN‐γ and resist production of IL‐17A is maintained in vivo and/or that receipt of YFP^+^ CD4^+^ T cells increases IFN‐γ production in the mice.

To confirm whether YFP^+^ naïve‐like CD4^+^ T cells remained committed to producing IFN‐γ in the T_H_17‐polarizing disease setting of the T‐cell transfer colitis model, we co‐transferred naïve‐like CD45.2^+^ YFP^+^ CD4^+^ T cells and naïve CD45.1^+^ CD4^+^ T cells (at a ratio of 1:9 within a total of 2,50,000 cells) into *Rag2^–/–^* mice. This ratio was chosen based on the previous in vitro experiment (Supporting Information Fig. S5B and C). The mice receiving cells lost weight after 7 weeks of transfer and had significantly larger colons and spleens compared to control *Rag2^–/–^* mice that did not receive a T cell transfer (Supporting Information Fig. S6A and B). The transferred YFP^+^ CD45.2^+^ population contained significantly more IFN‐γ single‐positive cells and significantly fewer IL‐17A single‐positive and IFN‐γ/IL‐17A DP cells, as expected, compared to the cotransferred CD45.1^+^ cells, which were able to produce IL‐17A (Fig. 5G and H, Supporting Information Fig. S6C). We conclude that YFP^+^ naïve‐like CD4^+^ T cells remain polarizsed to the T_H_1 lineage, even in *in vivo* in a T_H_1‐/T_H_17‐driven colitis model.

## Discussion

Here, we report the use of a novel T‐bet^FM^ mouse line to identify cells that have expressed the T_H_1 lineage‐specifying transcription factor T‐bet. The T‐bet^FM^ mouse line has allowed confirmation of known T‐bet expressing lineages and revealed previously uncharacterized T‐bet‐expressing cell populations. We identify a distinct subpopulation of CD4^+^ T cells that have a naïve surface phenotype and overall gene expression profile but that have experienced T‐bet expression. The cells arise after birth and rapidly upregulate IFN‐γ upon activation. The T_H_1 phenotype is stable both in repolarizing conditions *in vitro* and in a T_H_1‐/T_H_17‐driven inflammatory disease model *in vivo*. We conclude that T‐bet expression defines a population of T_H_1‐polarized naïve‐like cells that function to shape subsequent T_H_ cell responses.

The pattern of YFP positivity, marking cells that have experienced T‐bet expression, recapitulates previous knowledge of T‐bet function. For example, the expression of T‐bet in developing NKT cells and γδ T cells in the thymus [51, 52, 53, 54]. It has previously been reported that IFN‐γ‐producing γδ T cells express T‐bet and IL‐17A‐producing γδ T cells express RORγt [53]. However, our data suggest that, within the thymus, the subset of γδ T cells that will develop to become IL‐17A‐producing γδ T cells have also expressed T‐bet. Further phenotyping of these γδ T cells would be required to identify the different Vγ variable regions within these subsets [55]. As expected, developing thymic CD4^+^ SP, CD8^+^ SP, and DP cells were all YFP^–^negative. All cell types in the periphery that were expected to express T‐bet‐NK cells, CD4^+^ T_EM_ and T_CM_, and CD8^+^ T_EM_ and T_CM_ ‐ were positive for YFP [2, 3, 56]. Previous use of a different T‐bet fate mapping line (T‐bet‐ZsGreen‐T2A‐CreERT2‐Rosa26‐loxP‐STOP‐loxP‐tdTomato) identified populations of Treg and T_FH_ cells that had experienced T‐bet expression [57, 58]. These studies did not identify ZsGreen or tdTomato positive naïve CD4^+^ T cells, suggesting that the sensitivity of our model has allowed us to identify a hitherto unknown CD4^+^ T‐cell population.

Characterization of CD4^+^ T cells in our novel T‐bet fate‐mapping mouse line revealed a subset of cells that have naïve surface markers and a naïve‐like expression profile, but which have experienced T‐bet expression. This YFP‐positive population could be identified using a strictly defined set of naïve cell markers (CD62L^+^ CD44^–^ CD28^+^ CD27^+^ IL‐7R^+^ CD122^–^ CD95^–^). YFP^+^ naïve‐like cells also lacked markers of T_SCM_, T_MNP_, T_H_1‐like MP, or VM cell populations [40, 44–46, 59, 60]. Therefore, we suggest that these cells represent a previously unappreciated distinct population. YFP^+^ naïve‐like CD4^+^ cells were CD5^hi^, consistent with exposure to antigen, which is likely to be environmental. CD5^hi^ naïve cells have previously been shown to generate more MP cells than their CD5^lo^ counterparts [49], thus, we suggest that YFP^+^ naïve‐like cells may be precursors to T‐bet^hi^ MP cells. Experiments tracing the ontogeny of MP cells will be required to confirm this. Interestingly, 20% of YFP^+^ naïve‐like CD4^+^ T cells were positive for CXCR3, a T‐bet target gene that is required for the migration of both CD4^+^ and CD8^+^ T cells to sites of inflammation [5, 61‐63]. YFP^+^ CXCR3^+^ CD4^+^ T cells could be early immune responders that are able to migrate to areas of infection where they can rapidly induce a T_H_1 response. CXCR3 has previously been found to mark a population of human naïve CD8^+^ T cells with enhanced effector differentiation potential [64], suggesting parallels with the CD4^+^ population we have identified here. It has also been found that homeostatic expansion can drive naïve CD8^+^ T cells to take on a reversible memory‐like phenotype [65], which may also be related to the T‐bet‐experienced naïve CD4^+^ cell status we identify. It is also possible that naïve‐like YFP^+^ cells are at a very early state of T cell activation.

We found that YFP^+^ cells are absent from fetuses and only develop after birth, when they become detectable from as early as 1 week of age and remain throughout the lifespan of the animals. This suggests a requirement for an environmental antigen in the induction of T‐bet expression in these cells. This further supports the idea that T‐bet^+^ naïve‐like CD4^+^ T cells are early T_H_1‐polarized inflammatory responders that react to environmental antigens. Recent thymic emigrants (RTEs) have also been found to be CD31^+^ Qa2^lo^ CD24^hi^ and upregulate *Klf2* [66]. Determining whether YFP^+^ naïve‐like CD4^+^ T cells express these markers would be important to identify if these cells are RTEs. However, the use of a *Rag2p*‐GFP transgenic mouse would be needed to fully investigate this. On the other hand, it has been shown that CD4^+^ RTEs exhibit defective T_H_1 cell differentiation and express lower levels of T‐bet in comparison to mature naïve CD4^+^ T cells [67], suggesting that YFP^+^ naïve‐like cells may not be RTEs. Furthermore, the percentage of YFP^+^ naïve‐like CD4^+^ T cells remains constant, and even increases, in the spleen of older mice, despite age‐ related thymic involution and reduction in RTEs.

*In vitro* and *in viv*o analyses of YFP^+^ naïve‐like T cells show that they have a different phenotype to their YFP^–^ counterparts. The cells exhibit evidence of T_H_1 polarization and produce high amounts of IFN‐γ upon activation. YFP^+^ naïve‐like T cells also resist polarization to different lineages both *in vitro* and in a T_H_1‐/T_H_17‐driven colitis model *in vivo*. These data are again consistent with a role for these cells as early T_H_1‐polarized responders that produce IFN‐γ within inflammatory environments. These data also suggest that YFP^+^ naïve‐like T cells produce a more limited colitogenic phenotype due to their lack of IL‐17A production, which is required for induction of colitis in the naïve T‐cell transfer model [15, 24, 26‐28]. These data further emphasized that YFP^+^ naïve‐like CD4 T cells are committed to produce IFN‐γ, even in a T_H_17‐inducing inflammatory environment. T cells deficient in IFN‐γ production can still cause disease in the T‐cell transfer colitis model [68] and IFN‐γ has also been argued to be protective against T_H_17‐ /IL‐23‐mediated disease in this model [26, 69, 70]. The resistance of YFP^+^ cells to IL‐17A production, thus, correlates with the lower levels of colitis induced by naïve‐like YFP^+^ cells compared to their YFP^–^ counterparts.

The expression of T‐bet in cells with naïve surface markers suggests that lineage specification can proceed in the absence of canonical markers of T‐cell activation. Identification of signals responsible for T‐bet upregulation, and why these signals do not result in overt T cell activation, will require further investigation. The use of fate mapper mouse models to study the expression of other T cell lineage‐specifying factors will reveal whether this is a more general phenomenon.

In conclusion, we have identified a CD4^+^ T cell subset with a naïve‐like surface phenotype and expression profile that has experienced T‐bet expression, is polarized to the T_H_1 lineage, resists repolarization to other lineages, and which provides a rapid and stable source of IFN‐γ upon activation. This has implications for our understanding of the mechanisms that shape T‐helper responses in infection and inflammatory disease.

## Materials and Methods

### Animals

C56BL/6 *Rag1^–/–^* (Jackson labs) and *Rosa26^YFP/+^* (Jackson labs) mice were sourced commercially. *T‐bet^cre/+^* mice were previously generated by our group. *T‐bet^cre/+^* mice were bred with *Rosa26^YFP/+^* to generate the *T‐bet^cre/+^ Rosa26^YFP/+^* line (hereby referred to as T‐bet^FM^ mice). All mice used were aged between 6 and 12 weeks, unless stated otherwise. Fetal mice were culled at E.15.5 in accordance with humane Schedule 1 practice set out by the Home Office. All animal experiments were performed in accredited facilities in accordance with the UK Animals (Scientific Procedures) Act 1986 (Home Office Licence Numbers PPL: 70/6792 and 70/8127). T‐bet^FM^ mice were also housed and shipped from the University of Manchester.

### Cell isolation and preparation

Adult mice were euthanized using an approved Schedule 1 method of inhalation of a rising concentration of carbon dioxide gas followed by cervical dislocation. Embryo and neonatal mice were euthanized by using an approved Schedule 1 method of decapitation. Spleen, thymus, liver, mLN, peripheral (axillary, inguinal, and cervical) lymph nodes (pLN), and colons were excised and placed in cold PBS solution.

Colonic lamina propria cells were isolated as described [71]. Colons were cleaned, and fat and feces were removed. Afterwards, they were cut into 1–2 cm pieces using surgical scissors and put into 10 mL of HBSS without Mg^2+^/Ca^2+^ (Invitrogen) mixed with 5 mM EDTA and 10 mM HEPES (Fisher Scientific) and incubated at 37.5°C with agitation for 20 min. Next, intestinal pieces were filtered, and the subsequent intestinal pieces were sliced into fine pieces using scalpels and were collected in batch‐tested complete animal medium (RPMI [Gibco] with 10% heat‐inactivated FCS [Gibco], 2 mM glutamine, 100 U/mL penicillin, and 100 μg/mL streptomycin, HEPES (Fisher Scientific), nonessential amino acids, sodium pyruvate, and 2‐ME [Sigma]). Collagenase (0.5 mg/mL, Roche), DNase (10 μg/mL, Roche), and dispase II (1.5 mg/mL, Roche) were added and the intestinal pieces incubated for a further 20 min at 37°C with agitation. After incubation, the digestion mix was filtered once more and centrifuged at 860 *g* for 10 min at 4°C. Intestinal lamina propria cells were collected at the interface after centrifugation through Percol and washed.

Splenic, thymus, liver, mLN, and pLN cells were isolated into a single‐cell suspension in complete animal medium with the use of 70 μM mesh filter and general mechanical destruction. The suspension was centrifuged at 860 *g* for 5 min at 4°C and mLN and pLN, spleen, thymus, and liver cell pellets were resuspended, and red blood cells were lysed using a standard red blood lysis buffer (ACK). The liver pellet was then collected at the interface after centrifugation through Percol and washed.

### Cell sorting

CD4^+^ T cells from the spleen of mice were purified using LS positive selection MACs and anti‐CD4 (L3T4) beads (Miltenyi Biotec). CD4^+^ MACs sorted cells were further purified by FACS using a FACS Aria (BD Biosciences) with a 70 μm nozzle insert and FACS Diva software. CD4^+^ MACs sorted cells were stained for 20 min at 4°C in the dark with the following antibodies: anti‐CD4‐PerCPCy5.5 (RM4‐5), anti‐CD25‐PE (PC61), anti‐CD62L‐PECy7 (MEL‐14; Thermo Fisher), and anti‐CD44‐Pacific Blue (IM1.8.1; Thermo Fisher). Single‐positive compensation controls and unstained controls were used to set up instrument settings and for gating strategies. Cell purity was verified post‐sort (requiring 95% purification) and cell viability was assessed using trypan‐blue staining. Sorted cells were collected in complete media (as described above), counted using a hemocytometer, and immediately cultured or transferred in vivo.

### Naïve T cell transfer model of colitis

Naïve T cells were transferred as described [28]. Spleens and mLNs were harvested from either WT C56/BL6 mice or T‐bet^FM^ donor mice and mechanically disrupted. CD4^+^ T cells with naïve markers (CD4^+^ CD25^–^ CD44^low^ CD62L^high^) were sorted using a FACS Aria to a purity of >95%, washed, and resuspended in sterile PBS. *Rag2^–/–^* mice were injected intraperitoneally with 5 × 10^5^ CD4^+^ cells per mouse, unless stated otherwise, and humanely culled after 6–8 weeks. Mice were monitored for their health every week for signs of illness.

### Cell culture

Unfractionated single‐cell suspensions of splenocytes (2 × 10^6^/mL), mLN (1 × 10^6^/mL), and cLP cells (1 × 10^6^/mL) were cultured in complete animal medium (as described above) for 48 h in a CO_2_ controlled incubator at 37°C and 5% CO_2_.

In accordance with MIATA, sorted CD4^+^ T cells from the spleens of the T‐bet^FM^ mouse (as described above) were cultured at a concentration of 1 million cells per milliliters in complete media on preincubated anti‐CD3/anti‐CD28 bound plates in IL‐2 (20 ng/mL) (Sigma‐Aldrich) for 2 days and then just IL‐2 (20 ng/mL) (Sigma‐Aldrich) for a further 5 days. Supernatants were harvested and cytokine concentrations measured by ELISA (Thermo Fisher) and cells were stained and analyzed using flow cytometry as described below.

### CD4^+^ T cell polarization

Sorted CD4^+^ T cells from the spleens of the T‐bet^FM^ mouse (as described above) were cultured at a concentration of 1 million cells per milliliters in complete media on anti‐CD3‐/anti‐CD28‐bound plates for 2 days and specific cytokines added for either T_H_0: (hIL‐2 [20 ng/mL]), T_H_1: (anti‐IL‐4 [5 μg/mL], mIL‐12 [20 ng/mL], hIL‐2 [20 ng/mL]), T_H_2: (anti‐IFN‐γ [20 ng/mL], mIL‐4 [20 ng/mL], hIL‐2 [20 ng/mL]), T_H_17: (anti‐CD28 [5 μg/mL], anti‐IL‐4 [5 μg/mL], anti‐IFN‐γ [20 ng/mL], mIL‐1β [10 ng/mL], mIL‐6 [20 ng/mL], hTGF‐β [2 ng/mL]), and Treg: (hTGFβ [2 ng/mL], hIL‐2 [20 ng/mL]) polarizing conditions for a further 5 days. Supernatants were harvested and cytokine concentrations measured by ELISA (Thermo Fisher) and cells were stained and analyzed using flow cytometry as described below.

### *Ex vivo* **colon organ culture**

A total of 3‐mm punch biopsies (Miltex) were acquired from murine colons at full thickness. Three biopsies were cultured in 500 μL of complete animal RPMI for 48 h in a CO_2_ controlled incubator at 37°C and 5% CO_2_. Culture supernatants were harvested, and cytokine concentrations measured by ELISA (Thermo Fisher).

### ELISA

Cytokine concentrations for IL‐17A and IFN‐γ from the supernatants of cultured cells and *ex vivo* colon organ cultures were measured by ELISA (Thermo Fisher Ready‐Set‐Go). Samples and standards were measured in duplicate. Standard curves were created with the standards provided in the kits, in accordance with the manufacturer's protocols.

### Flow cytometry

Single‐cell suspensions extracted from the various tissues were plated out into flow cytometry tubes (Sarstedt) at a concentration of 10^6^ cells per milliliters. Cells were stimulated with 50 ng/mL phorbol 12‐myristate 13‐acetate (PMA) (Sigma Aldrich) and 1 μg/mL ionomycin (Sigma Aldrich) for 4 h. For intracellular staining, 2 μM monensin (Sigma Aldrich) was added for the final 2 h. For intracellular staining, cells were fixed and permeabilized using the Foxp3 fixation/permeabilization buffer kit (BD Biosciences). FcR receptor blocking antibodies were added for 15 min at 4°C and surface staining antibodies added together with live/dead stain (Fixable Live/Dead stains Thermo Fisher) and incubated for 20 min at room temperature in the dark. Cells were acquired within 24 h of staining. We used the following antibodies purchased from BD Biosciences, Biolegend, or Thermo Fisher: CD4 (RM4‐5), CD8‐α (53‐6.7), CD45.2 (104) CD45.1 (A20), CD3 (17A2), CD5 (53‐7.3), CD44 (IM7), CD62L (MEL‐14), CD25 (PC61), CD127 (A7R34), CD27 (LG.7F9), CD28 (37.51), CD49d (R1‐2), CD95 (15A7), CD11a (M17/4), CD122 (TM‐b1), CCR7 (4B12), CXCR3 (CXCR3‐173), T‐bet (4B10), RORγt (B2D), Foxp3 (FJK‐16S), GATA3 (LS0‐823), IL‐10 (JEs5‐16E3), IL‐5 (TRFK5), IL‐13 (eBio13A), IFN‐γ (XMG1.2), γδTCR (GL3), CD1d Tetramer (PBS57‐loaded or ‐unloaded, provided by NIH Tetramer Core Facility), B220 (RA3‐6B2), DX5 (DX5), CD11c (N418), CD11b (M1/70), Nkp46 (29A1.4), NK1.1 (PK136), c‐kit (ACK2), Sca‐1 (D7), hemotopoietic lineage cocktail (consisting of CD3 (17A2), B220 (RA3‐6B2), CD11b (M1/70), TER‐119 (TER‐119), Gr‐1 (RB6‐8C5), ICOS (C398.4A)). After incubation, cells were washed in sterile PBS and analyzed with a LSRFortessa flow cytometer (BD Biosciences). For flow cytometry set up and analysis, we adhered to the “Guidelines for the use of flow cytometry and cell sorting in immunological studies” [72].

### RNA sequencing (RNA‐seq)

YFP^+^ and YFP^–^ naïve‐like (live CD3^+^ CD4^+^ CD62L^+^ CD44^–^) and effector memory (live CD3^+^ CD4^+^ CD62L^–^ CD44^+^) CD4^+^ T cells were sorted by FACS. Cells were sorted into 1 mL of lysis buffer and either immediately processed for RNA extraction or frozen at −80°C for RNA extraction later using the RNAeasy Micro kit (Qiagen). Purified RNA was checked for the quality, contamination, and concentration using a NanoDrop, Qubit spectrophotometer, and Agilent Bioanalyzer. Libraries were prepared using the Ovation SoLo RNA‐seq library preparation kit (NuGEN genomics) according to the manufacturer's instructions and were sequenced on an Illumina HiSeq 3000 to generate 2 × 75 bp paired‐end reads. Reads were filtered to remove adaptors and low‐quality bases and aligned to mm9 using TopHat2 [73]. PCR duplicates were removed using the NuGEN duplicate marking tool and read counts generated using featureCounts [74]. Differential gene expression analysis between YFP^+^ and YFP^–^ naïve or YFP^+^ and YFP^–^ T_EM_ CD4^+^ T cells was conducted using DEseq2 [75], and gene expression levels across all samples were calculated and normalized using the regularized logarithm transformation [75]. GSEA was performed using the fgsea package using 1000 permutations [76] with a T_H_1 gene signature identified from three different T_H_1 versus T_H_2 gene expression datasets [77, 78, 79]. Fastq files were downloaded from the SRA and gene‐centred expression estimates made using kallisto together with the Gencode M20 transcript models. T_H_1‐specific genes were then identified using DESeq2, with study and cell type (T_H_1/T_H_2) treated as covariates for batch correction.

### Statistical analysis

Statistical analyses were carried using GraphPad Prism 8 (GraphPad Software Inc.). Significance was calculated using the Mann‐Whitney U test or, for grouped data, the two‐way ANOVA test with Dunn's corrections. Alpha was set at 0.05.

## Conflict of Interest

The authors declare that there is no conflict of interest.

## Author Contributions

Study concept and design (JWL, GML, RGJ) data acquisition (JWL, MVdM, LBR), data analysis and interpretation (JWL, MVdM, SH, LBR, JFN, NGM, ES), technical support (LBR, NGM, AH, JFN, ES, IJ, LC, ELH, ASD), obtaining funding (RGJ, GML, JKH, JFN, NP), and study supervision (RGJ, GML). The manuscript was written by JWL and edited by RGJ, and was critically reviewed by all authors.

### Peer review

The peer review history for this article is available at https://publons.com/publon/10.1002/eji.202149228.

**Abbreviations**
APCs: antigen presenting cells
cLP: colonic lamina propria
DCs: dendritic cells
DN: double‐negative
DP: double‐positive
γδ T: gamma delta T
GSEA: gene set enrichment analysis
IFN‐γ: interferon‐gamma
IL: interleukin
mLN: mesenteric lymph nodes
MP: memory‐phenotype cell
NK: natural killer
NKT: natural killer T
pLN: peripheral lymph nodes
pTreg: peripheral regulatory T cells
Rag: recombinant activation gene
RTEs: recent thymic emigrants
SP: single‐positive
STAT: Signal transducer and activator of transcription
T‐betFM: T‐bet fate mapping
TCM: central memory T cells
TCR: T cell receptor
TEM: effector memory T cells
TFH: follicular helper T cells
TH: helper T cells
TMNP: memory T cells with a naïve phenotype
Treg: regulatory T cells
TSCM: T‐memory stem cells
VM: virtual memory

## Supporting information

Suporting information

TableS1

## Data Availability

The data that support the findings of this study are openly available at Gene Expression Omnibus (GEO) with accession code GSE153805. This can be accessed with the secure token enufyogsxrutzut.
