# Supplementary material for: A population of naive‐like CD4+ T cells stably polarized to the TH1 lineage
Source: Eur J Immunol. 2022 Feb 12;52(4):566–81. doi: 10.1002/eji.202149228 (PMC9304323; doi:10.1002/eji.202149228)
Supplement: Supplementary file 1 — Suporting information [file EJI-52-566-s001.pdf]

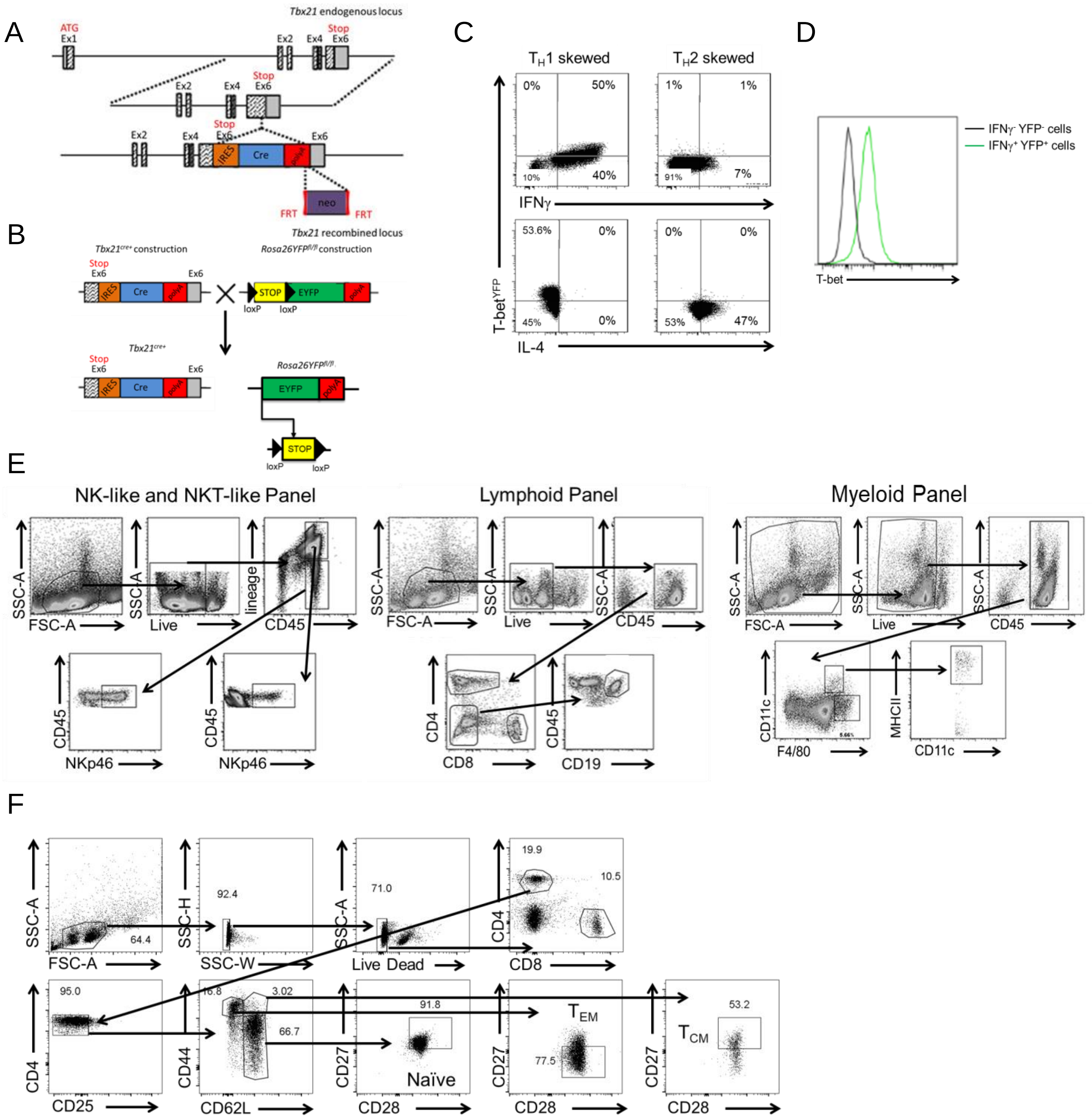

Supplemental Figure 1. Characterising YFP expression in the T-bet^{FM} mouse.

A. Schematic showing the insertion of Cre into exon 6 of *Tbx21*.

B. Schematic showing the generation of the T-bet^{cre} x ROSA26YFP$^{fl/fl}$ T-bet^{FM} mouse line.

C. Representative flow plots showing cytokine and YFP expression in naïve (CD62L^{+} CD44^{-}) CD4^{+} T-cells after activation with anti-CD3/CD28 and polarisation in T$_{H}$1 and T$_{H}$2 conditions (from 1 independent experiments with an n of 2).

D. Representative histogram showing T-bet expression in IFNγ^{+} YFP^{+} and IFNγ^{-} YFP^{-} cells in after T$_{H}$1 polarisation, as shown from C.

E. Representative flow plots showing the gating strategy for NK-like and NKT-like cells, lymphoid cells and myeloid cells.

F. Representative flow plots showing the gating for CD4^{+} T-cell subsets in the spleen. This strategy was also used for all organs.

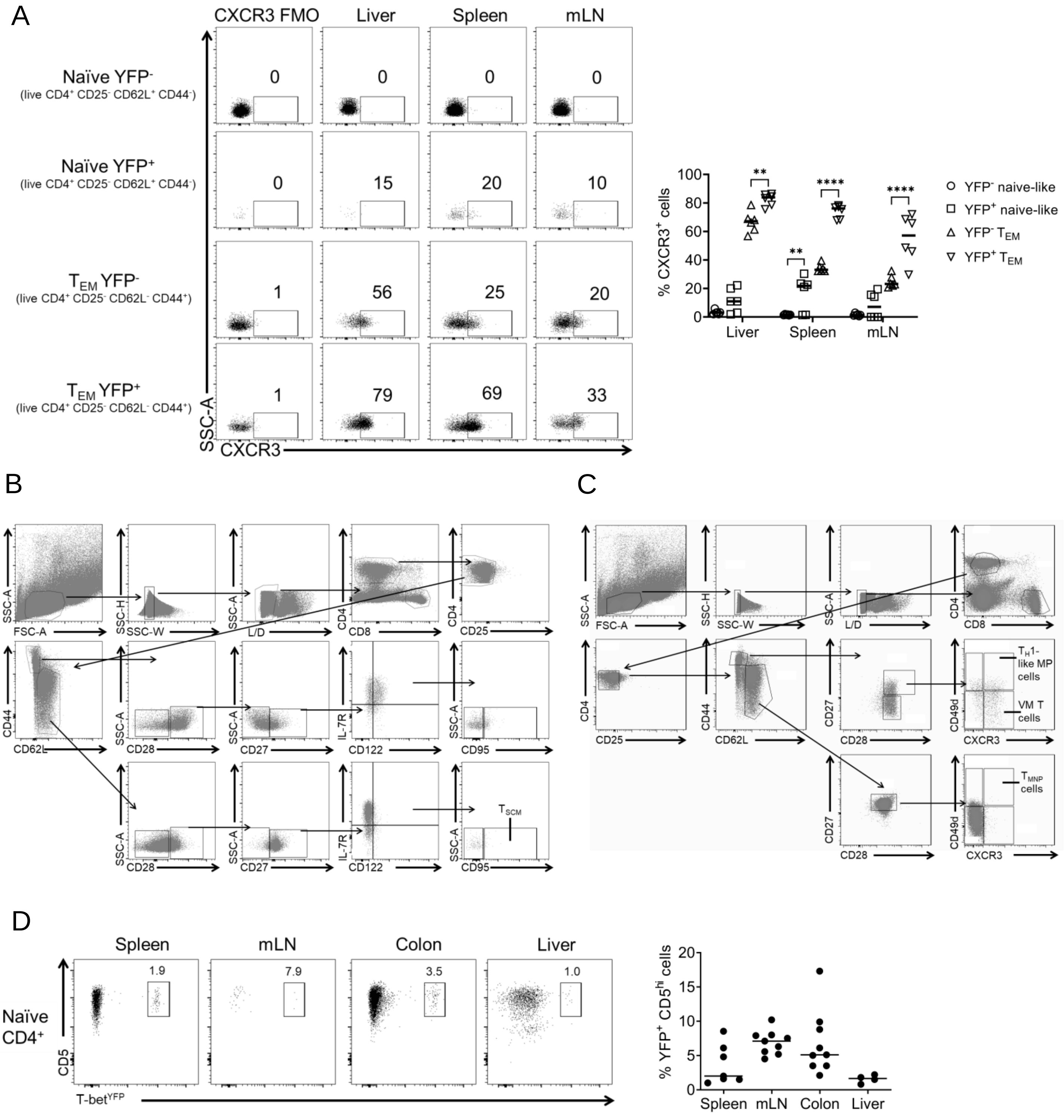

Supplemental Figure 2. Analysis of T_{SCM}, VM, T_H1-like MP, T_{MNP} $CD4^+$ T cells and $CD5^{hi}$ MP cells.

A. Representative flow plots and median proportions of $CXCR3^+$ $CD4^+$ T cells in the YFP^- or YFP^+ naïve ($CD62L^+$ $CD44^-$) or YFP^- or YFP^+ memory ($CD44^+$ $CD62L^-$) compartments from spleen, mLN, colon and liver (n=6, from two independent experiments), ** $p<0.01$ **** $p<0.0001$ (Two-way ANOVA).
B. Gating strategy for identifying T_{SCM} in the naïve ($CD62L^+$ $CD44^-$) compartment in the spleen.
C. Gating strategy for identifying T_{MNP}, T_H1-like MP and VM T cells in the naïve ($CD62L^+$ $CD44^-$) or memory ($CD44^+$ $CD62L^-$) compartments in the spleen.
D. Representative flow plots and mean proportions of naïve YFP^+ $CD5^{hi}$ $CD4^+$ T cells (live $CD3^+$ $CD4^+$ $CD62L^+$ $CD44^-$) in the spleen, mLN, colon and liver (n=6 for spleen and mLN, n=5 for colon and n=4 for liver, from two independent experiments).

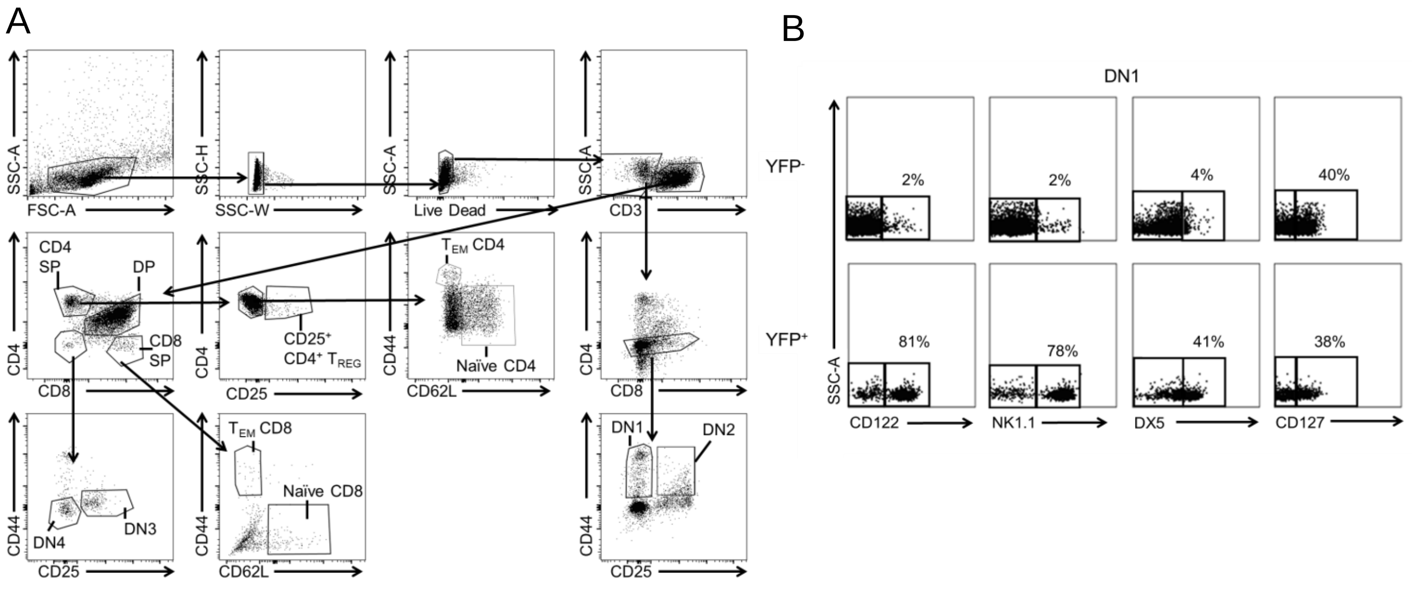

Supplemental Figure 3. YFP expression in T cells of the thymus.

A. Representative flow plots showing the gating strategy used for cells from the thymus.

B. Representative flow plots showing the proportion of YFP$^-$ and YFP$^+$ DN1 cells with NK and NKT markers.

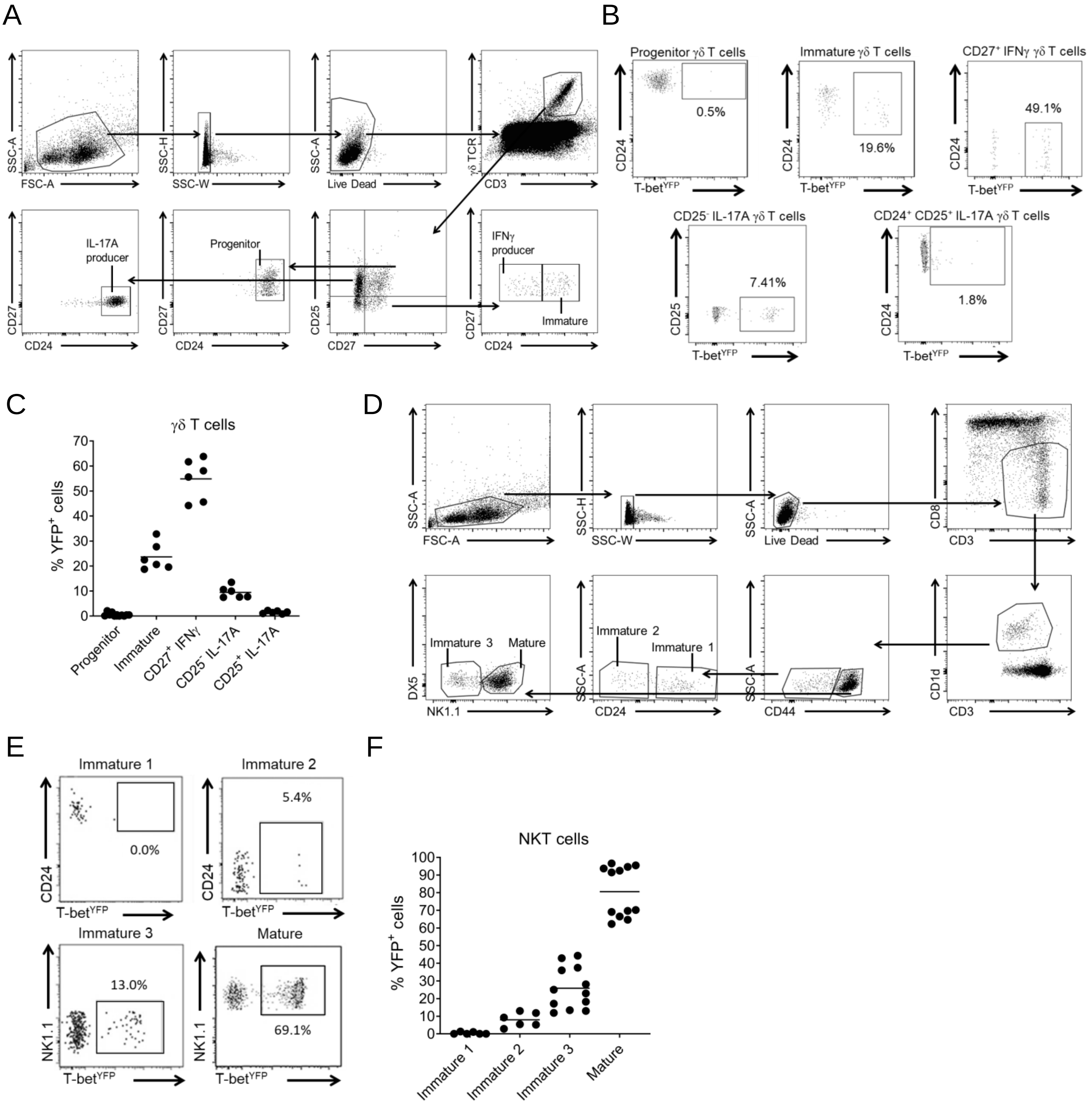

Supplemental Figure 4. YFP expression in γδ T and NKT cells in the thymus.

A. Representative flow plots showing the gating strategy used to identify γδ T cells in the thymus.

B. Representative flow plots showing YFP expression in five populations of developing γδ T cells in the thymus: progenitor γδ T cells ($CD25^+$ $CD27^+$ $CD24^+$), immature γδ T cells ($CD25^-$ $CD27^+$ $CD24^+$), IFNγ-producing γδ T cells ($CD25^-$ $CD24^-$ $CD27^+$) and the two sets of IL-17A-producing γδ T cells ($CD25^-$ $CD27^-$ $CD24^-$ and $CD25^+$ $CD27^-$ $CD24^+$).

C. Mean proportions of YFP^+ cells from the different stages of γδ T cell development shown in B (n=6).

D. Representative flow plots showing the gating strategy used to identify NKT cells in the thymus.

E. Representative flow plots showing YFP expression in the four populations of developing NKT cells in the thymus: Immature 1 ($CD44^-$ $CD24^+$), immature 2 ($CD44^-$ $CD24^-$), immature 3 ($CD44^+$ $CD24^-$ $NK1.1^-$) and mature NKT cells ($CD44^+$ $NK1.1^+$).

F. Mean proportions of YFP^+ cells from the different stages of NKT cell development shown in E (n=12).

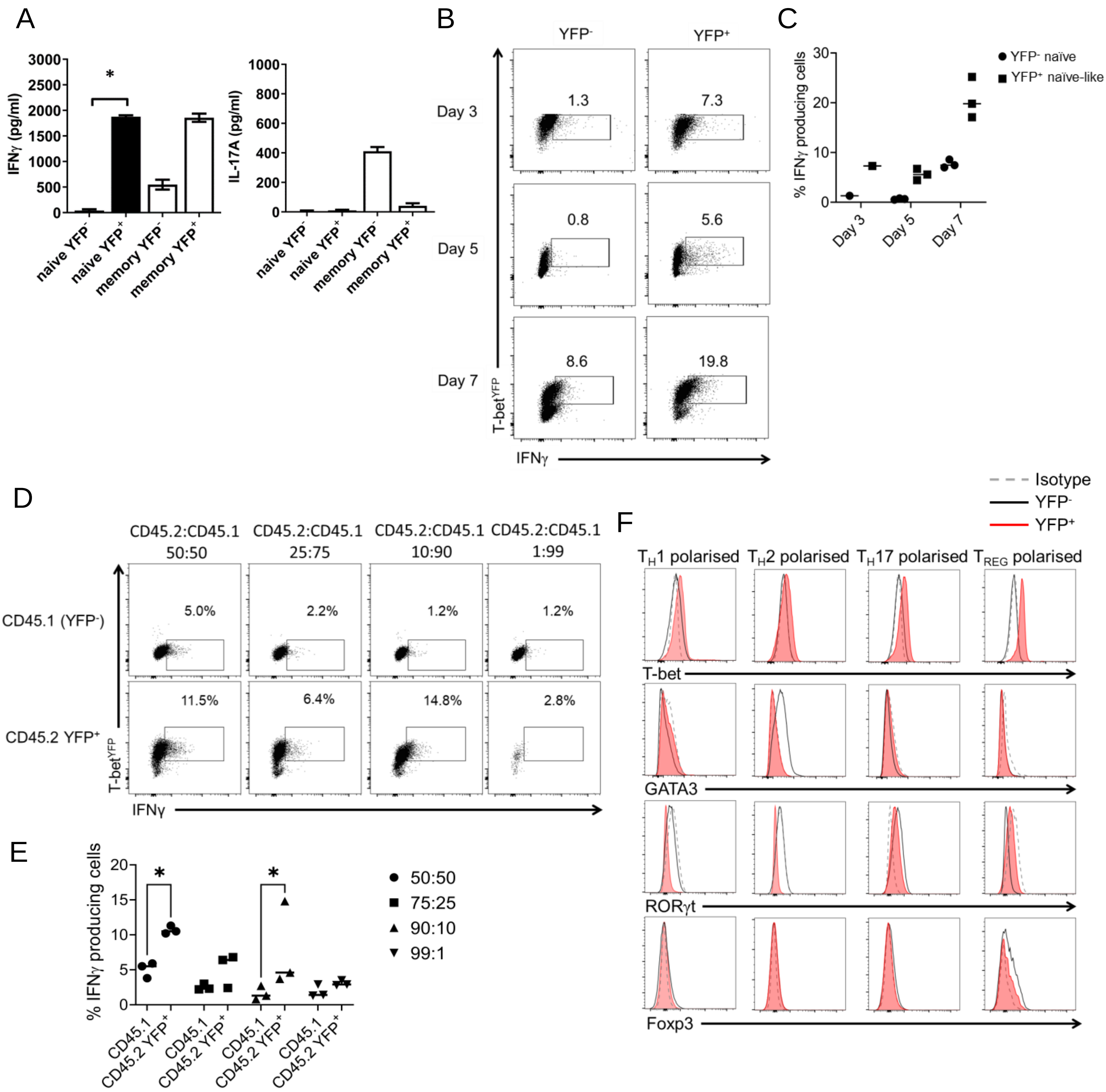

Supplemental Figure 5. YFP+ naïve-like CD4+ T cells are predisposed to produce IFNγ upon activation.

A. IFNγ and IL-17A in the supernatant of cultured cells measured by ELISA (n=6, except n=2 for YFP+ naïve-like CD4+ T cells, * P<0.05 (Kruskal-Wallis test with Dunn's corrections)).
B. Representative flow plots showing YFP and IFNγ expression by naïve-like (CD62L+ CD44-) YFP- and YFP+ CD4+ T cells after activation with anti-CD3/CD28 and culture with IL-2 for 3, 5 or 7 days.
C. Mean proportions of IFNγ-producing CD4+ T cells shown in B (n=3 for days 5 and 7 and n=1 for day 3, each with 3 technical replicates).
D. Representative flow plots showing the percentage of IFNγ-producing CD4+ T cells after 7 days of *in vitro* co-culture of CD45.1 and CD45.2 YFP+ CD4+ T cells at different ratios in only IL-2.
E. Mean proportions of IFNγ-producing CD4+ T cells shown in D (n=3, * p<0.05 (Kruskal-Wallis test)).
F. Representative histograms showing transcription factor expression in naïve-like CD4+ T cells after polarisation into different lineages (n=3 biological replicates, each with 3 technical replicates).

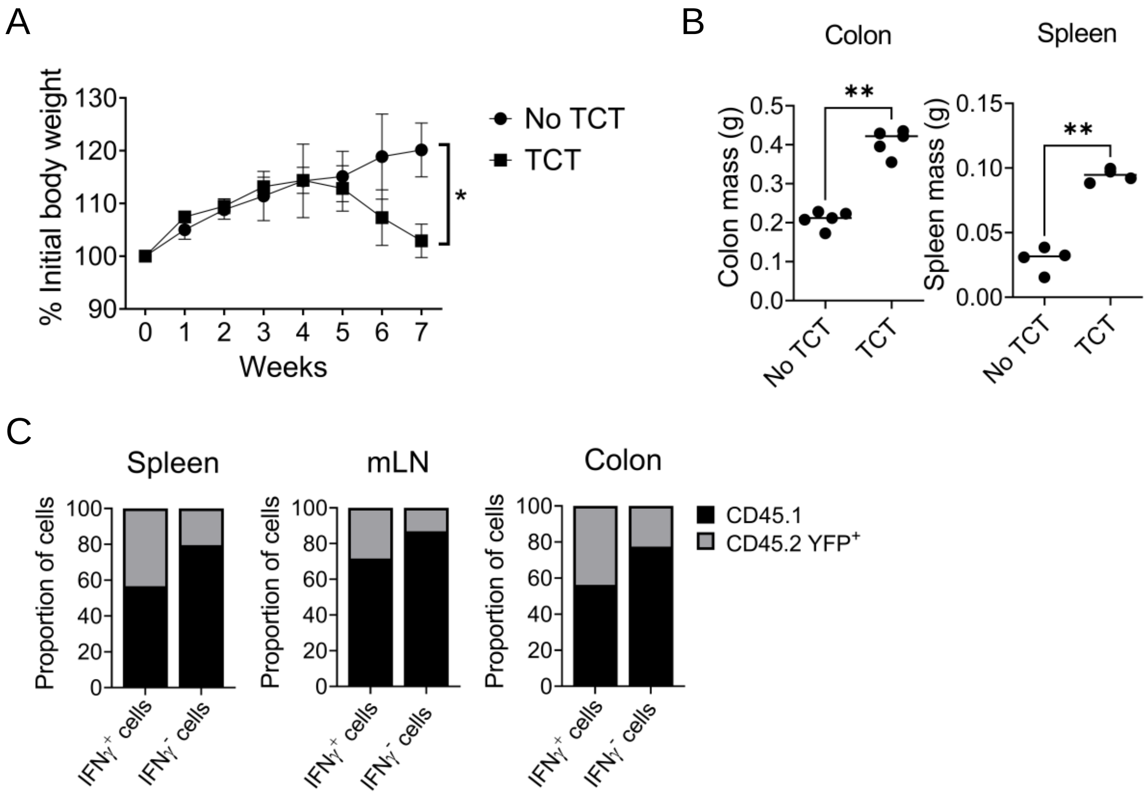

Supplemental Figure 6. CD45.2 YFP$^+$ CD4$^+$ T cells remain polarised to T$_H$1 phenotype despite co-transfer to a colitis model.

A. Mean weight of *Rag2$^{-/-}$* mice after co-transfer of 250,000 naïve-like (CD62L$^+$ CD44$^-$) CD45.1 and CD45.2 YFP$^+$ CD4$^+$ T-cells at a ratio of 9:1 (n=8) compared with *Rag2$^{-/-}$* without T cell transfer (TCT) (n=5), two independent experiments, * $p<0.05$ (multiple Mann-Whitney U test, statistical significance shown for week 7).

B. Mean spleen and colon mass in *Rag2$^{-/-}$* mice after co-transfer of 250,000 naïve-like (CD62L$^+$ CD44$^-$) CD45.1 and CD45.2 YFP$^+$ CD4$^+$ T-cells at a ratio of 9:1 (n=5) compared with *Rag2$^{-/-}$* mice without TCT (n=5) ** $p<0.01$ (Mann-Whitney U test).

C. Percentages of CD45.1 and CD45.2 cells in the populations of IFNγ$^+$ and IFNγ$^-$ producing cells from the CD45.2 YFP$^+$/CD45.1 co-transfer experiments (n=10).
